# Intermedin protects against renal ischemia-reperfusion injury by inhibiting endoplasmic reticulum stress

**DOI:** 10.1186/s12882-015-0157-7

**Published:** 2015-10-23

**Authors:** Yanhong Wang, Jihua Tian, Xi Qiao, Xiaole Su, Yang Mi, Ruijing Zhang, Rongshan Li

**Affiliations:** Department of Microbiology and Immunology, Shanxi Medical University, Taiyuan, Shanxi China; Department of Nephrology, Second Hospital of Shanxi Medical University, Taiyuan, Shanxi China; Department of Nephrology, the Affiliated People’s Hospital of Shanxi Medical University, Shanxi Provincial People’s Hospital, Shanxi Kidney Disease Institute, No. 29 Shuang Ta East Street, Taiyuan, 030012, Shanxi P. R. China

**Keywords:** Intermedin, Renal, Ischemia-reperfusion injury, Endoplasmic reticulum stress, Apoptosis

## Abstract

**Background:**

Intermedin (IMD) is a novel member of the calcitonin/calcitonin gene-related peptide family. Endoplasmic reticulum stress (ERS) has been implicated in the pathology of renal ischemia/reperfusion (IRI). In the present study, we investigated whether IMD could reduce ERS damage after renal ischemia.

**Methods:**

The kidneys of SD rats were subjected to 45 min of warm ischemia followed by 24 h of reperfusion. The hypoxia/reoxygenation(H/R) model in NRK-52E cells consisted of hypoxia for 1 h and reoxygenation for 2 h. IMD was over-expressed in vivo and in vitro using the vector pcDNA3.1-IMD. The serum creatinine concentration and lactate dehydrogenase (LDH) activity in the plasma were determined. Histologic examinations of renal tissues were performed with PAS staining. Real-time PCR and Western blotting were used to determine the mRNA and protein levels, respectively. Additionally, ER staining was used to detect the ERS response.

**Results:**

In the rat renal IRI model, we found that IMD gene transfer markedly improved renal function and pathology and decreased LDH activity and cell apoptosis compared with the kidneys that were transfected with the control plasmid. IMD significantly attenuated the ERS stress parameters compared with IRI group. Indeed, IMD down-regulated glucose-regulated protein 78 (GRP78), C/EBP homologous protein(CHOP), and caspase 12 protein and mRNA levels. Moreover, in the NRK-52E cell H/R model, IMD overexpression prevented the apoptosis induced by H/R. Furthermore, IMD ameliorated the ER structural changes and concomitantly decreased the levels of GRP78, CHOP and caspase-12.

**Conclusion:**

This study revealed that IMD protects against renal IRI by suppressing ERS and ERS-related apoptosis.

## Background

Renal ischemia-reperfusion injury (IRI) is a general health problem and the most common cause of acute kidney injury (AKI) [1–3]. IRI affects both native and transplanted kidneys and is associated with high morbidity and mortality [4]. Evidence has shown that many factors, including reactive oxygen species (ROS), abnormal protein synthesis, Ca^2+^ overload and apoptosis, are involved in the development of renal IRI [5].

Several studies have evidenced the pivotal role of endoplasmic reticulum stress (ERS) as a major contributor to the increase in apoptosis and the exacerbation of cell damage after IRI [6, 7]. The ER is the principal site of protein synthesis and folding, Ca^2+^ storage, and signaling. Various stimuli, such as ER Ca^2+^ overload, elevated protein synthesis, ischemia, and hypoxia, interact with ER homeostasis and result in ERS [8]. Moreover, excessive and/or prolonged ERS triggers the apoptotic death program [9]. ERS is considered to be an early or initial response of cells that are stressed or damaged and occurs extensively in the pathophysiology of renal IRI [10–12]. Thus, ERS causes cell damage and apoptosis, but the inhibition or remodeling of the ERS response pathways may provide a new therapeutic intervention strategy for renal H/R and IRI injury.

Intermedin/adrenomedullin-2 (IMD/ADM2) is a novel member of the calcitonin gene-related peptide (CGRP) family and is distributed in a wide variety of tissues, including brain, heart, lung, gastrointestinal tract, pituitary, and kidney tissues [13, 14]. IMD is considered to be potential endogenous protective agent in the heart, vascular system, and kidneys [15, 16]. Our previous investigations demonstrated that IMD gene transfer significantly reduces renal ischemia/reperfusion injury by inhibiting oxidative stress, ameliorating inflammation and promoting renal cell proliferation and regeneration [17, 18]. It has been reported that IMD can protect against myocardial IRI by inhibiting myocardial ERS, and the phosphoinositide 3-kinase/Akt(PI3K/Akt) signaling pathway is involved in this inhibitory effect [19]. However, the detailed mechanism by which IMD protects against renal IRI and the association of this protective effect with ERS have not been investigated. Therefore, the aim of this study was to investigate whether IMD protects against renal IRI by inhibiting ERS in incubated rat tubular epithelial cells in vitro and in a rat renal IRI model.

## Methods

### Ultrasound-mediated gene delivery into the kidney

The eukaryotic expression plasmid pcDNA3.1-IMD containing full-length cDNA sequence of rat IMD was created according to the methods described in our previous study [20]. Ten minutes after the removal of the right kidney, the pcDNA3.1-IMD plasmid or an empty control vector(pcDNA3.1) was transfected into the left kidney via the renal artery using an ultrasound-mediated system as described in our previous study [18].

### Animals

All male SD rats (180–200 g) were purchased from the Experimental Animal Center of Shanxi Medical University (Taiyuan, China) and maintained in a specific pathogen-free environment in our facility. All animals were fed with standard chow and had free access to water. All animal experiments were performed in a humane manner, and also in accordance with the Institutional Animal Care Instructions. This study was conducted under experimental protocols approved by the Ethics Committee for Animals, Shanxi Medical University.

### Renal IRI model and experiment design

The animals were randomly divided into the following four groups: sham, IRI, IRI + IMD, and IRI + empty plasmid. The renal IRI model was created by clamping the left renal artery with a non-traumatic vascular clamp for 45 min 1 week after the removal of the right kidney. The clamp was then removed, the kidney was observed for the return of blood flow, and the incision was sutured. The sham rats were underwent a similar procedure without the occlusion of the renal artery. The IRI + IMD rats and IRI + empty plasmid rats were treated with ultrasound as described above 1 week before renal IRI. The rats were killed 24- h after reperfusion. Blood and kidney samples were collected and submitted to the corresponding examinations.

### Measurements of renal function and LDH activity

Renal function was assessed by measuring the serum creatinine concentrations, and the LDH activity was assessed with a commercially available colorimetric method (Nanjing Jiancheng Bioengineering Institute, Nanjing, P.R. China). These experiments were performed at least three times.

### Histological examination

After fixation with 10 % paraformaldehyde, paraffin-embedded transverse kidney slices were sectioned at 3 μm and stained with Periodic Acid-Schiff (PAS) stain. The histopathological scoring was performed by an experienced pathologist who was unaware of the treatment. The scores were given based on gradings of the tubular necrosis, nuclear pyknosis, cast formation, and tubular dilatation and interstitial infiltration in the outer medulla of 10 randomly chosen, non-overlapping fields (400×), and the mean of all of the scores was used for the comparisons between the groups. The morphologic changes were scored semiquantitatively on a 0 to 5+ scale (0, no lesion; 1+, <10 % of parenchyma affected by the lesion; 2+, 11–25 % of parenchyma affected by the lesion; 3+, >26–45 % of parenchyma affected by the lesion; and 4, >46–75 % of parenchyma affected by the lesion,; and 5, >76 % of parenchyma affected by the lesion).

### TUNEL assay

To detect tubular cell apoptosis, a terminal deoxynucleotidyl transferase dUTP nick-end labeling (TUNEL) apoptosis detection kit (Roche, Mannheim, Germany) was used. Briefly, each slide was deparaffinized and rehydrated, and treated with proteinase K (20 mg/L) for 15 min. The endogenous peroxidase was inhibited with 3 % hydrogen peroxide for 5 min, and then incubated with the TUNEL reaction mixture containing terminal deoxynucleotidyl transferase (TdT) and digoxigenin-11-dUTP for 1 h. The TdT reaction was carried out in a humidified chamber at 37 °C, and then 3,3-diaminobenzidine chromogen was applied. Hematoxylin was used as counterstaining. Apoptotic cell death was quantitatively analyzed by counting the TUNEL-positive cells selected randomly from 10 fields, at 400× magnification. The percentages of TUNEL-positive cells were calculated and averaged.

### Cell culture and treatments

The rat tubular epithelial cell line NRK-52E was obtained from the Cell Bank of the Chinese Academy of Sciences (Shanghai, China). The cells were cultured in DMEM/Ham’s F-12 (2:1) containing with 100 u/ml penicillin—streptomycin, 10 % fetal bovine serum and incubated at 37 °C, room air with 5 % CO2 . The cells were seeded in 6-well plates until they reached approximately 80 % confluence. The over-expression of IMD in the NRK-52E cells that were transfected with pcDNA3.1-IMD was accomplished according to the methods described in our previous study [20]. The model of H/R injury in the NRK-52E cells was established in an incubator as described previously [21]. In brief, the cells were washed with DMEM/F12 and then incubated in modified Tyrode’s solution [22] with 95 % N2 + 5 % CO2 for 1 h (hypoxia). Reoxygenation was achieved by switching the medium to fresh DMEM/F12, and the cells were subsequently maintained in 95 % air-5 % CO_2_ for 2 h. The cells were assigned to 1 of 5 groups: control (untreated NRK-52E cells); tunicamycin (Tm) (NRK-52E cells were incubated with Tm (10 mg/ml, Sigma, USA) for 30 min); H/R (the cells were exposed to 1 h of hypoxia and 2 h of reoxygenation); H/R + pcDNA3.1 (NRK-52E cells were transfected with the empty plasmid pcDNA3.1 prior to H/R); and H/R + IMD (IMD was overexpressed in NRK-52E cells exposed to H/R).

### Determination of apoptosis in NRK-52E cells

The apoptosis of the NRK-52E cells was measured by flow cytometry (FCM) with a FITC Annexin V Apoptosis Detection Kit I (BD Pharmingen ^TM^, New Jersey, USA) according to the manufacturer’s instructions. The apoptosis ratio (%) was the ratio of the sum of the cells that stained positive for Annexin V-FITC plus the cells that stained positive for both Annexin V-FITC and PI divided by the entire population of cells.

### ER staining

The cells were plated on glass-bottom culture dishes and subjected to different treatments. The ER staining was performed according to the protocol from the kit (GMS10041.2, Genmed, Arlington, MA). The presence of ER staining was visualized under a confocal immunofluorescence microscope (Olympus, Japan).

### Semiquantitative RT-PCR

Total renal RNA was extracted with Trizol reagent (Invitrogen, Carlsbad, CA). First-strand cDNA was synthesized using oligo dT primers and reverse transcription was performed according to the manufacturer’s instructions using M-MLV reverse transcriptase (TaKaRa Biotechnology Co., Ltd., Dalian, China) with total RNA as a template. RT-PCR was performed using a RT-PCR Kit following the manufacturer’s instruction (TaKaRa Biotechnology Co., Ltd., Dalian,China). The 25 -μl reaction volume containing 2.5 μl 10 × PCR buffer, 4.0 μl dNTP, 1.0 μl each primer, 0.25 μl Taq polymerase and 2.0 μl cDNA mix. PCR was initiated with a denaturation step at 94 °C for 3.0 min, followed by 30 cycles of amplification (94 °C for 30 s, 54 °C for 30 s, and 72 °C for 1.0 min), and a final step at 72 °C for 10 min. The PCR produced was separated using 1.0 % agarose gel. The specific primers were used for rat IMD (sense: 5′-GGAAATCGTGCGTGACATTAAG-3′ and antisense: 5 ′-GGACTCGTCATACTCCTGCTTG-3′) and β-actin (sense: 5′-CCTCACTTCGGCCTGTAGTT-3′ and antisense: 5′ -ACCCACCTCAGCCATAACTT-3′).

### Reverse transcription polymerase chain reaction and real-time polymerase chain reaction

Total RNA was isolated using Trizol (TAKARA, Dalian, China). Complementary DNA (cDNA) was obtained from total the RNA using the SuperScript First-Strand synthesis system RT-PCR kit (TAKARA, Dalian, China). qPCR amplifications were performed in triplicate using the SYBR Green I assay and the Stratagene M3000 Sequence Detection System (Stratagene, USA). The gene sequences were identified in GenBank to design the specific primers (Table 1). The reactions were performed in 96-well plates in 20-μl reaction volumes in the following conditions: 95 °C for 5 min, followed by 40 cycles of 95 °C for 5 s and 60 °C for 30 s. In each assay, a standard curve was determined concurrently with the examination of the samples. Gene expression was quantified using a modification of the 2-ΔΔct method. All PCR reactions were performed in triplicate.

**Table 1:** Primers for real-time PCR

Gene	Primer sequences(5′ to 3′)	
GRP78	Forward	5-GCATCCTGGTGGCTTTCCAGCCATTC-3
	Reverse	5-CTGGGTACATTTGATCTGACTGG-3
CHOP	Forward	5-CTGCTCCTTCTCCTTCATGC-3
	Reverse	5-AGCAGAGGTCACAAGCACCT-3
caspase12	Forward	5-TCCCTTTGCTTGTGGATACC-3
	Reverse	5-GAAGGAATCTGTGGGGTGAA-3
β-actin	Forward	5-TGGCTCCTAGCACCATGAAG-3
	Reverse	5-GCTCAGTAACAGTCCGCCTAGA-3

### Western blot analysis

The cortical tissues of the rats’ kidneys and cell lysates were extracted in lysis buffer (KeyGEN Biotech, Nanjing, China). Equal amounts of protein (50 μg) as determined using a BCA assay kit (KeyGEN Biotech, Nanjing, China) were separated on 10 % SDS-polyacrylamide gels and electrophoretically transferred to a nitrocellulose filter membrane (Millipore, Bedford, MA). After incubation with 5 % bovine serum albumin (BSA, dissolved in PBS) for 2 h at room temperature and blocking of the non-specific binding, the membranes were incubated overnight at 4 °C with primary antibodies (Santa Cruz Biotechnology, Santa Cruz, CA) at dilution of 1:1000–5000. Then, the fluorescein-linked secondary antibody (Santa Cruz Biotechnology, Santa Cruz, CA) was added at a dilution of 1:3000 to the membrane and incubated at room temperature for 2 h. The specific bands were visualized by fluorography using an enhanced chemiluminescence kit (Pierce, Rockford, USA). The relative density was quantified using the Quantity One analysis system (Bio-Rad, California, USA).

### Statistical analysis

The data are expressed as the means ± the SDs. Significant differences between groups were assessed by one-way ANOVA with Student-Newman-Keuls post hoc tests. *P* values < 0.05 were considered statistically significant.

## Results

### The transfection efficiency of IMD by ultrasound-mediated gene delivery into the Kidney in vivo and by FuGENE HD into NRK-52E cells in vitro

**Fig. 1:**
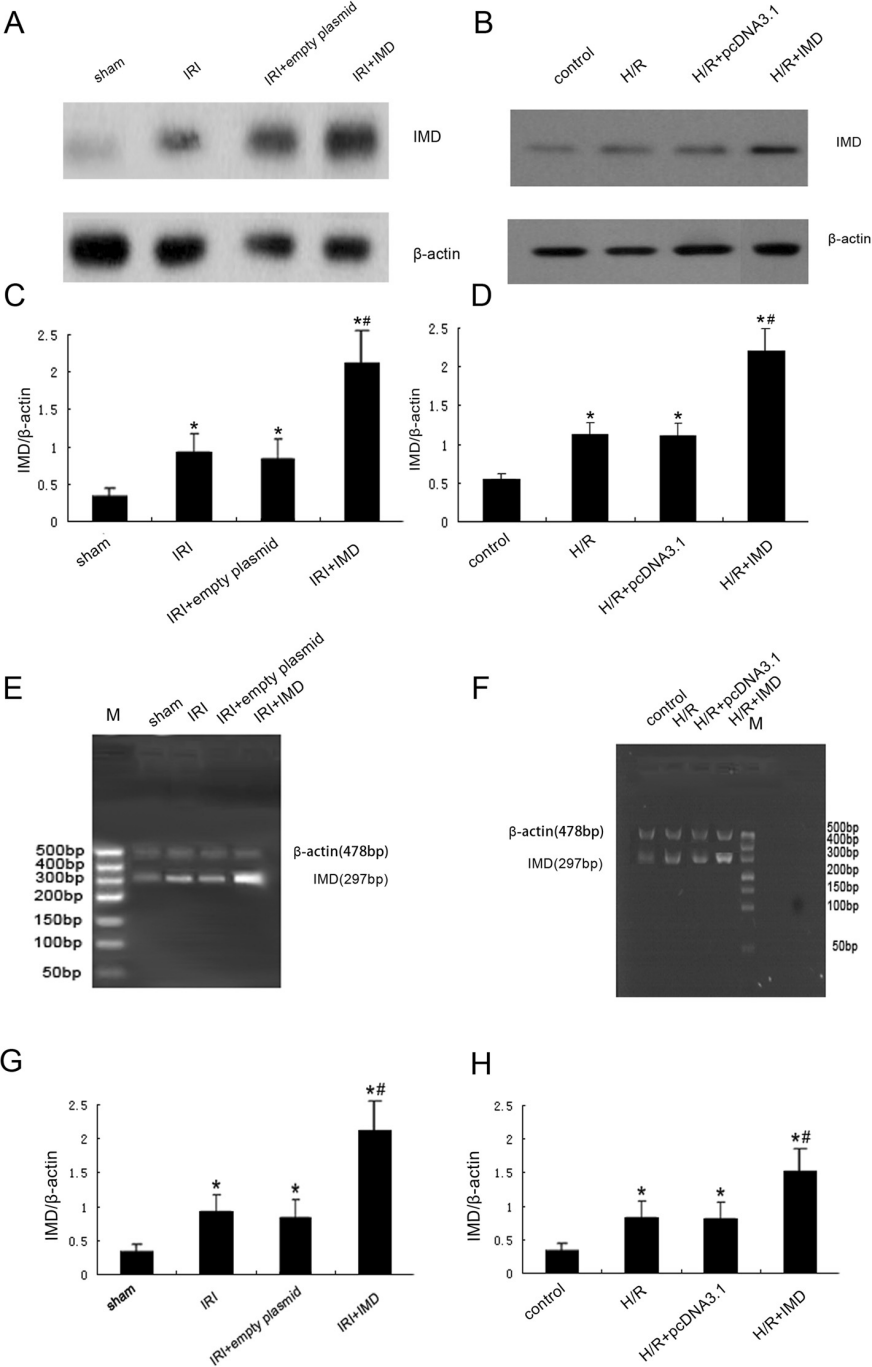
The transfection efficiency of IMD by ultrasound-mediated gene delivery into the Kidney in vivo and by FuGENE HD into NRK-52E cells in vitro. **a** Representative IMD protein expression measured by Western blot in rats, (**b**) representative IMD protein expression measured by Western blot in NRK-52E cells, (**c**) Quantitative analysis of IMD by Western blots in rats, (**d**) Quantitative analysis of IMD by Western blots in NRK-52E cells, (**e**) representative IMD mRNA expression measured by RT-PCR in rats, (**f**) representative IMD mRNA expression measured by RT-PCR in NRK-52E cells, (**g**) densitometric quantifications of band intensities from RT-PCR for IMD/β-actin in rats, (**h**) densitometric quantifications of band intensities from RT-PCR for IMD/β-actin in NRK-52E cells. M: DL500 DNA marker, Data in bar graphs are means ± SD, *n* = 6.* *P* < 0.05 vs. sham group or control group; # *P* < 0.05 vs. IRI group or H/R group, respectively

To determine the efficacy of ultrasound-microbubble-mediated gene transfection in kidney and by FuGENE HD in NRK-52E cells in vitro, we examined the expression of IMD in kidneys and NRK-52E cells with semiquantitative RT-PCR and Western blot analysis. As shown in Fig. 1, after 7 days of transfection, kidneys from rats treated with pcDNA3.1-IMD plasmid exhibited significant increase in IMD expression compared with kidneys of rats treated with control empty vector , and the expression of IMD in NRK-52E cells transfected with pcDNA3.1-IMD plasmid was much high than in controls, indicating that IMD was transfected into the kidney and NRK-52E cells successfully.

### IMD ameliorated the renal injury in the rats after IRI treatment

**Fig. 2:**
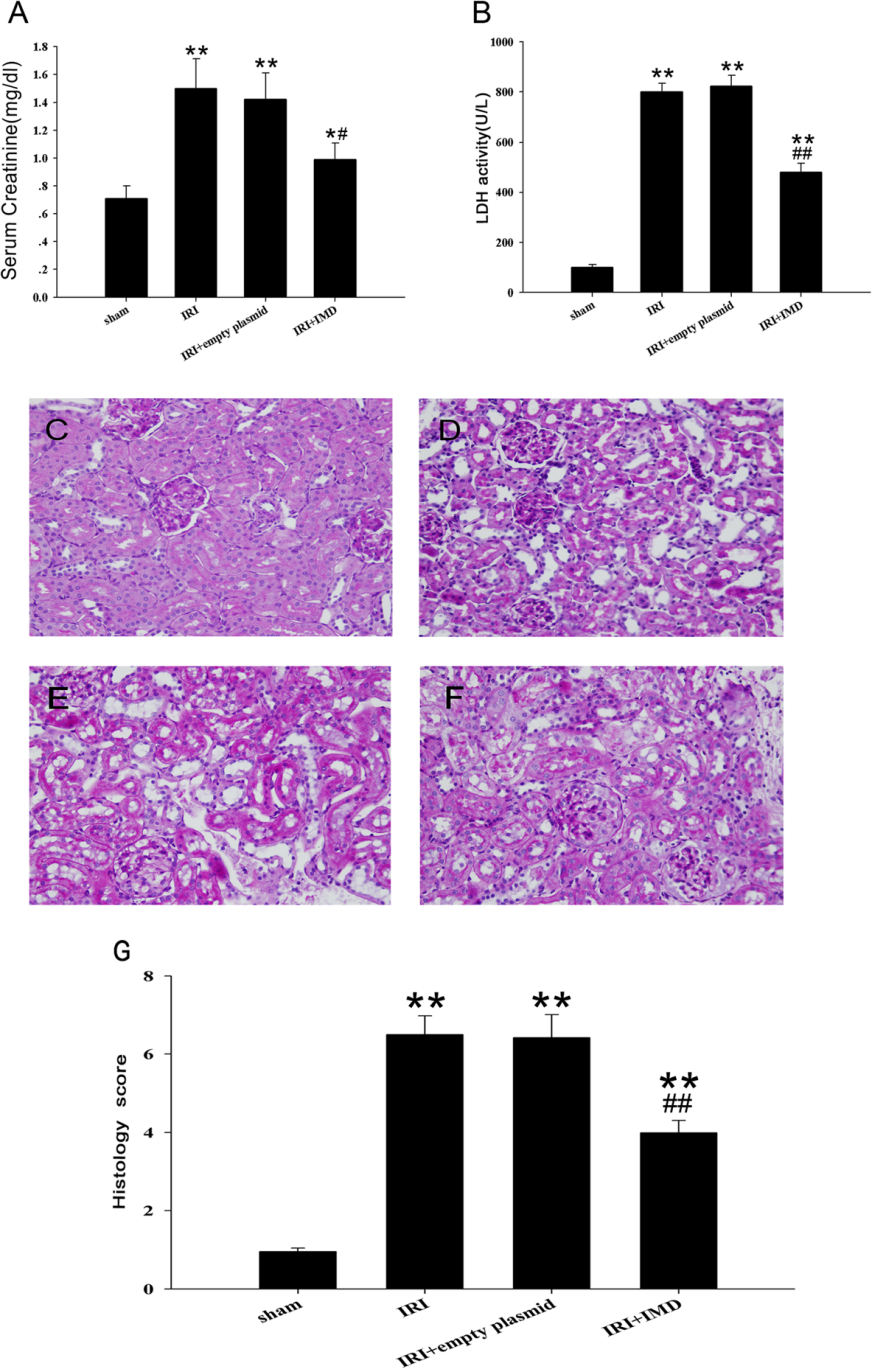
IMD ameliorated renal injury in rats after IRI treatment. **a** Serum creatinine concentration, (**b**) the LDH activity in different IRI treatment groups. **c** - **f** Renal pathomorphological changes after renal ischemia-reperfusion injury (IRI), Original magnification, ×400; (**c**) Sham groups, (**e**) IRI groups, (**e**) IRI + empty plasmid groups, (**f**) IRI + IMD groups, Magnification:×400 (**g**) semiquantitation of the morphological changes by histological grading system. Data in bar graphs are means ± SD, *n* = 8. * *P* < 0.05, **P < 0.01 vs. sham group; # *P* < 0.05, ## *P* < 0.01 vs. IRI group, respectively

As shown in Fig. 2, renal IRI resulted in a significant increases in plasma Cr levels (1.52 ± 0.24 mg/dl l vs. 0.71 ± 0.09 mg/d, *P* < 0.01) and LDH activity (805.17 ± 35.12 U/L vs. 114.21 ± 12.15 U/L, *P* <0.01) compared with the sham group. The over-expression of IMD elicited significant improvements in cells integrity and reductions in renal functional injury compared with the IRI group (*P* <0.05). On light microscopy, the PAS staining revealed that there were severe pathological changes in the kidneys with renal IRI that included a loss of the brush-border membranes, tubular dilatation, flattened tubular epithelia, cast formation, luminal debris, and interstitial infiltration (Fig. 2d). In contrast, the overexpression of IMD significantly attenuated these pathological renal abnormalities (Fig. 2f). In the kidneys transfected with the empty vector, the levels of serum Cr, LDH and the pathological changes exhibited no significant differences compared with the kidneys of the non-transfected IRI group. This result indicated that the improved kidney function and renal pathological changes in rats was due to the overexpression of IMD.

### IMD suppressed cell death triggered by renal IRI

**Fig. 3:**
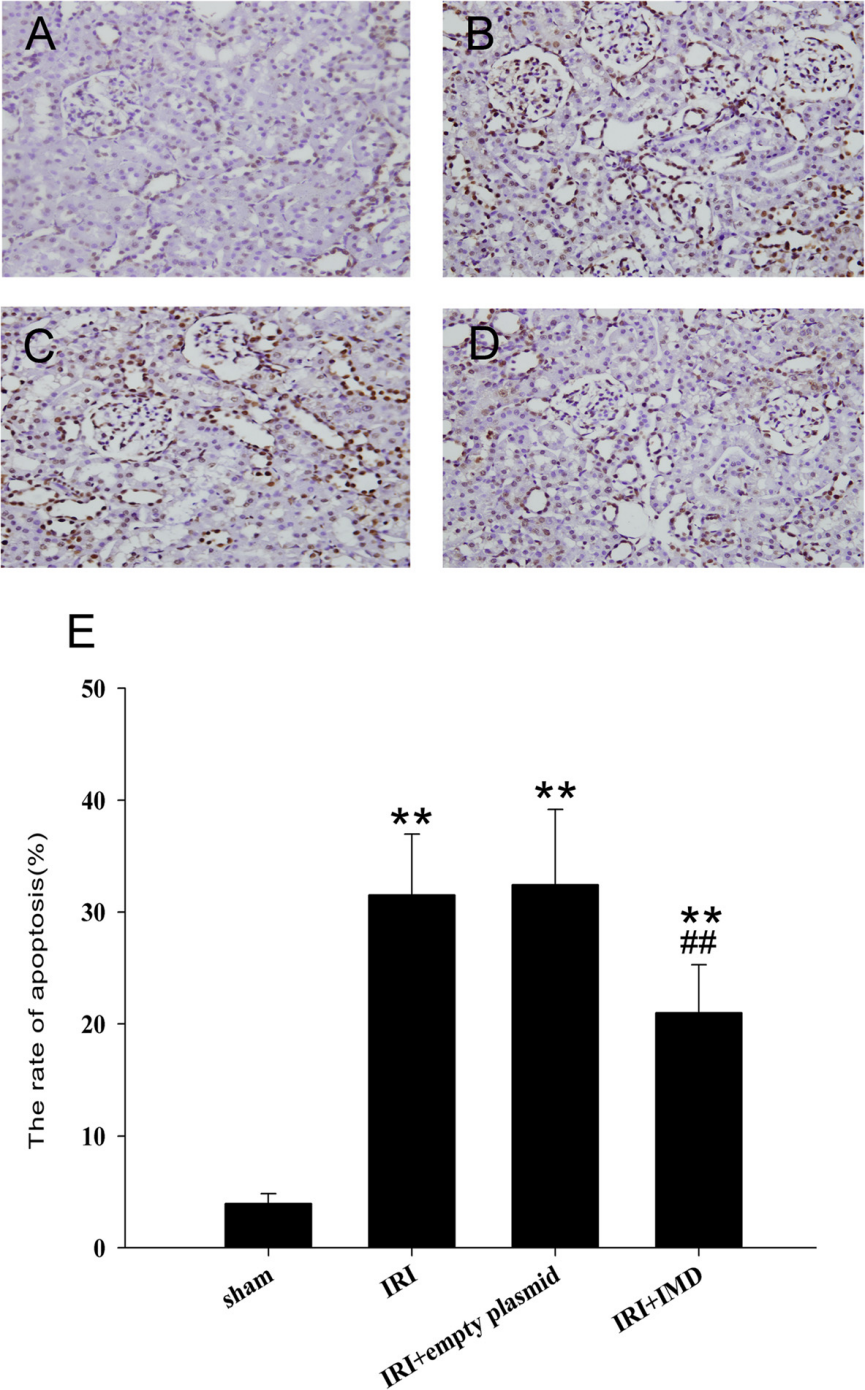
IMD suppressed cell apoptosis triggered by renal IRI. **a** - **d** Determination of tubular cell apoptosis in situ by terminal deoxynu-cleotidyl transferase biotin-dUTP nick end-labeling (TUNEL) assay. (×200) (**a**) Sham groups, (**b**) IRI groups, (**c**) IRI + empty plasmid groups, (**d**) IRI + IMD groups, Magnification:×400 (**e**) Determination of positive tubular cells in the TUNEL assay. Data in bar graphs are means ± SD, *n* = 8. **P < 0.01 vs. sham group; ## *P* < 0.01 vs. IRI group

The cell death in the rat kidneys following IRI were assessed using a TUNEL assay. Our results indicated that the TUNEL‑positive renal tubular epithelial cells were significantly more numerous in the IRI group than in the sham group (31.5 ± 5.46 % vs. 3.95 ± 0.89 %, *P* < 0.01, Fig. 3). However, a significant reduction in the number of death tubular cells in the kidney was detected in the IRI + IMD group (20.99 ± 4.32 %). These results suggested that IMD suppressed tubular cell death and thereby protected the kidney from renal IRI.

### IMD reduced ERS stimulated by renal IRI

**Fig. 4:**
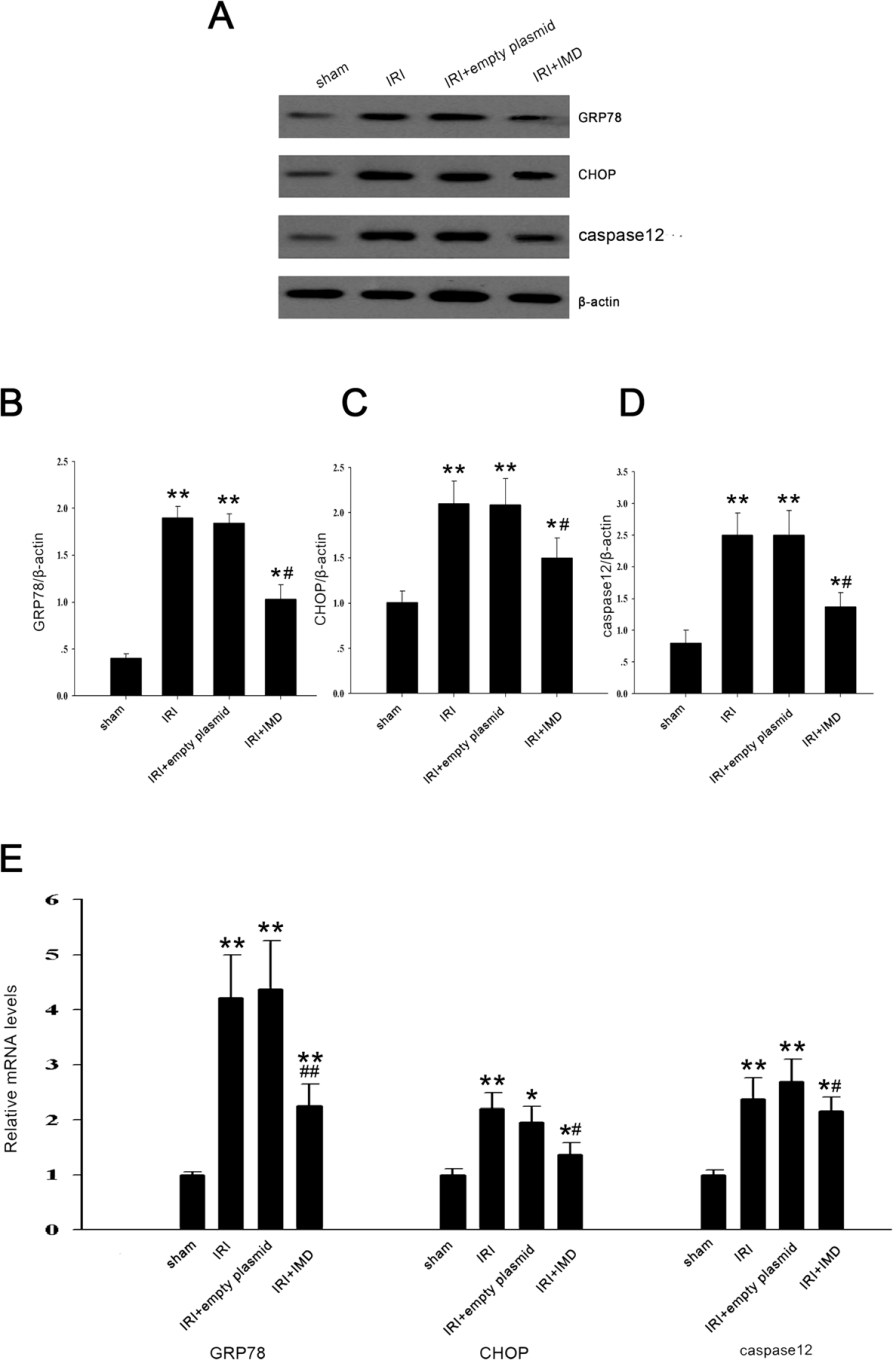
Effects of IMD on markers of endoplasmic reticulum stress (ERS) in IRI rats. **a** Representative protein expression of GRP78, CHOP, caspase-12, and β-actin as a control; Quantitative analysis of (**b**) GRP78, (**c**) CHOP, and (**d**) caspase-12 by Western blots. **e** The mRNA expression levels of GRP78, CHOP and caspase-12 were determined by real time-PCR. Data in bar graphs are means ± SD, *n* = 8. * *P* < 0.05, **P < 0.01 vs. sham group; # *P* < 0.05, ## *P* < 0.01 vs. IRI group, respectively

Several studies have evidenced the pivotal role of ERS as a major contributor to the increase in apoptosis and the exacerbation of cell damage following IRI. We performed western blot and realtime PCR and found that renal IRI caused a robust enhancements of GRP78, CHOP, and caspase 12, compared with the sham group(all *P* <0.01, Fig. 4), and these changes were indicative of ERS. The overexpression of IMD significantly reduced the GRP78 level (*P* < 0.05). Similarly, this treatment significantly reduced the relative amounts of CHOP and caspase 12 (*P* < 0.05) compared to IRI alone. As a control, the empty plasmid did not improve the ERS parameters compared to IRI group; the parameters of both of these groups remained high compared to those of the sham group (*P* <0.01). These results suggested that the protective effects of IMD may have been mediated by the repression of ERS.

### IMD inhibited ERS-related apoptosis in the NRK-52E cells with H/R injury

**Fig. 5:**
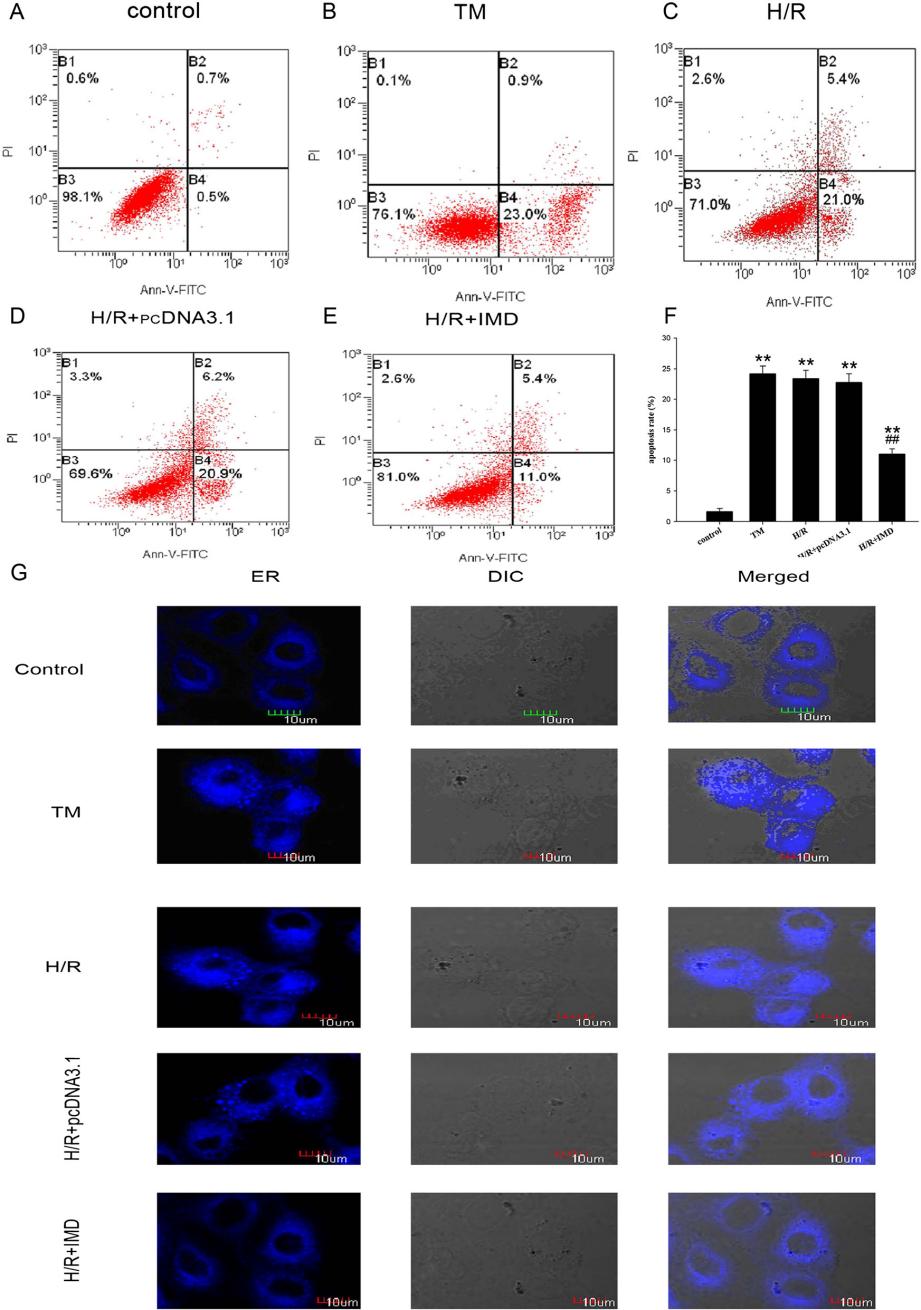
IMD inhibited ERS-related apoptosis in NRK-52E cell of H/R injury. **a** - **e** Cell apoptosis measured by flow cytometry. **a** control, **b** Tm, **c** H/R, **d** H/R + pcDNA3.1, **e** H/R + IMD, **f** The apoptosis ratio in different H/R treatment groups. **g** ER morphology (blue) in NRK-52E cells loaded with Dapoxyl according to the kit. Data in bar graphs are means ± SD, *n* = 6. **P < 0.01 vs. control group; ## *P* < 0.01 vs. Tm or H/R group, respectively

To determine whether IMD had a direct inhibitory effect on renal ERS, the ERS-inducer Tm (10 mg/ml) was incubated with the rat tubular epithelial NRK-52E cells line in vitro. We assessed cell apoptosis using a flow cytometry assay. As shown in Fig. 5a - f, the apoptosis activity ratios were significantly higher in H/R and Tm groups than in the control group. The ER staining in the fresh viable NRK-52E cells (Fig. 5g) demonstrated that the structure of the ER was severely destroyed in the NRK-52E cells subjected to H/R treatment. The fluorescence density revealed a non-uniform distribution that was accompanied by notable vacuoles in the ER. Moreover, there were no significant differences between the H/R and Tm groups in any of these results. Therefore, we suggest that H/R and Tm were equally effective in inducing ERS-related apoptosis. Compared to the H/R group, the over-expression of IMD significantly reduced the number of apoptotic tubular cells among the NRK-52E cells. ER staining also demonstrated that the IMD treatment significantly attenuated the injury to the ER.

**Fig. 6:**
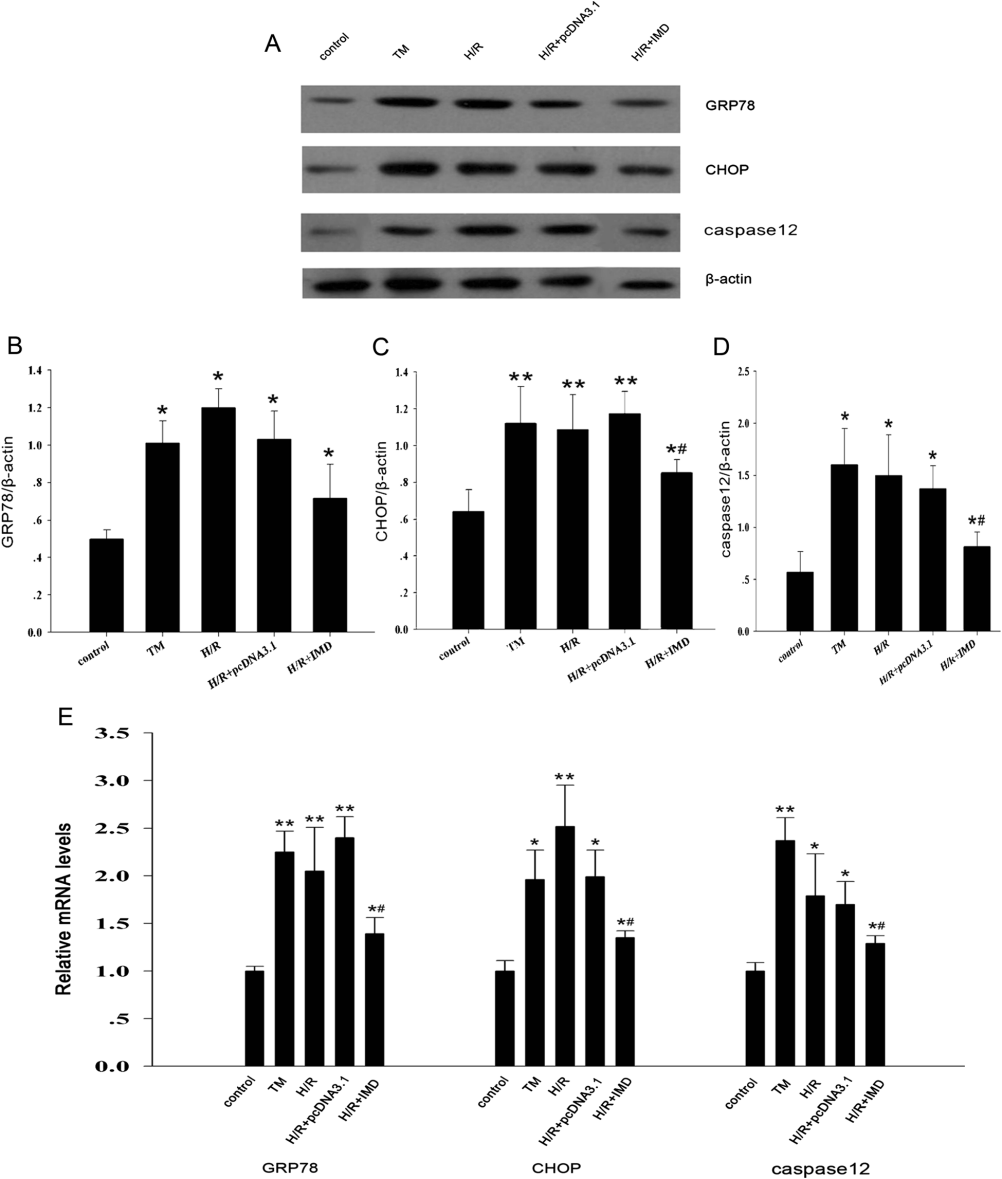
IMD inhibited tunicamycin (Tm) and H/R-induced (ERS) in vitro. **a** Representative protein expression of GRP78, CHOP, caspase-12, and β-actin as a control; Quantitative analysis of (**b**) GRP78, (**c**) CHOP, and (**d**) caspase-12 by Western blots. **e** The mRNA expression levels of GRP78, caspase-12, CHOP were determined by real time-PCR. Data in bar graphs are means ± SD, *n* = 6. * *P* < 0.05, **P < 0.01 vs. control group; # *P* < 0.05 vs. Tm or H/R group, respectively

Compared with the control group, H/R and Tm treatment significantly upregulated the protein expressions of GRP78, CHOP, and caspase-12 (all *P* < 0.05) and IMD attenuated the upregulated expression of the H/R-induced overexpressions of the ERS marker proteins (all *P* < 0.05; Fig. 6a–d). The mRNA expressions of GRP78, CHOP and caspase-12 confirmed these results (Fig. 6e).

## Discussion

In the present study, we showed that the overexpression of IMD can ameliorate the renal dysfunction and protect against the renal damage caused by IRI. Moreover, IRI can up-regulate the expression of ERS markers, such as GRP78, CHOP and caspase 12, and induce ERS-related apoptosis. Our data suggest that the protective effects of IMD in renal IRI were primarily mediated through the inhibition of ERS and ERS-related apoptosis.

Intermedin (IMD), also called adrenomedullin 2 (ADM-2), is a novel member of the calcitonin gene-related peptide (CGRP) superfamily [13, 14] and has been shown to have pathophysiological effects in multiple disease processes involving the circulatory and renal systems [15, 16, 23, 24]. Recently, we showed that IMD gene transfer significantly reduces renal ischemia/reperfusion injury apparently by reducing oxidative stress and promoting renal tubular cell proliferation and regeneration after renal cell H/R injury [17, 18].

Our results revealed that the overexpression of IMD in the kidney significantly improved renal function, protected against the renal damage caused by IRI, and attenuated renal pathological abnormalities. Moreover, the results of our TUNEL assay revealed that the rate of apoptosis among the renal tubular cells was lower in the IMD + IRI group than in the IRI group. These results suggest that IMD protected the kidneys of the rats in the IRI model by improving their structures and functions and inhibiting cell apoptosis.

Renal tubule epithelial cell apoptosis induced by IRI is the main cause of the development and progression of AKI [25, 26]. Some recent evidence strongly suggests that renal IRI induces ERS and that the involvement of ERS is an initiator of cell death during ischemic glomerular and tubular epithelial injury following renal IRI [27, 28]. The ER is one of the largest organelles in the cell and plays an important role in the regulation of cellular homeostasis. The ER is responsible for the synthesis, modification, and processing of proteins, the chain folding, assembly, and transportation of new peptides, the biosynthesis of steroids, cholesterol, and many lipids, and intracellular calcium regulation. However, several insults can interfere with this machinery and cause aberrant protein folding. The accumulation of unfolded or misfolded proteins in the ER lumen induces ERS, which in turn activates a well-conserved adaptive response [29–31]. However, when ERS is excessive or severe, these adaptive responses are not sufficient to relieve the ERS, and cell apoptosis is induced through the activation of a signaling pathway, such as the C/EBP homologous protein (CHOP), caspase-12, or JNK signaling pathway [32, 33]. In the physiological state, glucose-regulated protein 78 (GRP78) binds to the effectors of UPR to suppress their activities [34]. Under conditions of ER stress, when misfolded proteins accumulate in the ER lumen, GRP78 dissociates from these effectors and allows their activations [35]. Under physiological conditions, these mediators are inactivated by binding to GRP78 in the ER membrane, and GRP78 dissociates from these effectors and allows their activations [36]. When IRI-induced ERS is too severe to overcome, UPR gradually develops into the apoptotic phase. CHOP accumulation, IRE1 phosphorylation and JNK activation initiate the apoptotic phase and finally lead to cell apoptosis in renal IRI [37–40]. Thus, GRP78, CHOP and caspase-12 are generally considered to be strong markers of the ERS level [19].

To confirm the effect of IMD on ERS, we detected the levels of these markers of ERS (i.e., GRP78, CHOP and caspase-12) in a rat renal IRI model. The results revealed that the mRNA and protein expression levels of GRP78, CHOP and caspase-12 in the kidney were significantly increased in the IRI rats and that IMD treatment significantly inhibited the increased levels of the renal ERS factors and ERS-induced renal injury and apoptosis. Additionally, we used the specific ERS inducer Tm [41] to induce ERS in vitro in the rat tubular epithelial cell line NRK-52E. Our flow cytometry assay and ER staining results confirmed that the apoptosis rates were elevated and that the structure of the ER was severely destroyed in the NRK-52E cells in the Tm and in vitro H/R groups. Moreover, the overexpression of IMD attenuated the rates of apoptosis and improved the ER structures. Moreover, the Tm and H/R groups also exhibited upregulations of the ERS markers GRP78, CHOP and caspase-12, and IMD inhibited these upregulations. Therefore, the protective effects of IMD against renal injury were mediated, at least in part, by the inhibition of renal ERS.

## Conclusion

Our study findings indicate that the overexpression of IMD in the kidney protects against renal IRI apparently by reducing ERS and consequently suppressing ERS-induced apoptosis. Thus, IMD might be a new target for the inhibition of ERS and the improvement of renal function in ischemia-mediated acute renal failure.

## Abbreviations

ADM: Adrenomedullin
AKI: Acute kidney injury
cDNA: Complementary DNA
CGRP: Calcitonin gene-related peptide
CHOP: C/EBP homologous protein
ER: Endoplasmic reticulum
ERS: Endoplasmic reticulum stress
FCM: Flow cytometry
FITC: Fluorescinisothiocyate
GRP78: Glucose-regulated protein 78
H/R: Hypoxia/reoxygenation
IMD: Intermedin
IRE-1: Inositol requiring enzyme-1
IRI: Ischemia/reperfusion
JNK: cJUN NH2-terminal kinase
LDH: Lactate dehydrogenase
PAS: Periodic acid schiff
PI3K/AKT: Phosphoinositide 3-kinase/Akt
ROS: Reactive oxygen species
Tm: Tunicamycin
TUNEL: Terminal deoxynucleotidyl transferase dUTP nick end labeling
UPR: Unfolded protein response
